# Detection Rate of Fetal Anomalies in Early Mid-Trimester Compared to Late Mid-Trimester Detailed Scans: Possible Implications for First-Trimester Sonography

**DOI:** 10.3390/jcm13195750

**Published:** 2024-09-27

**Authors:** Zangi Yehudit, Michaelson-Cohen Rachel, Weiss Ari, Shen Ori, Mazaki Eyal, Sela Hen Yitzhak

**Affiliations:** 1 Department of Obstetrics & Gynecology, Shaare Zedek Medical Center, Faculty of Medicine, Hebrew University School of Medicine, Jerusalem 9112102, Israel; ylazikin@gmail.com (Z.Y.); rachelmc@szmc.org.il (M.-C.R.); 2 Medical Genetics Unit, Shaare Zedek Medical Center, Faculty of Medicine, Hebrew University School of Medicine, Jerusalem 9112102, Israel; ariweiss10@gmail.com (W.A.); eyalmazaki@hotmail.com (M.E.)

**Keywords:** anomalies, screening, ultrasound, mid-trimester, pregnancy

## Abstract

**Objective**: A late mid-trimester fetal organ scan (lMTS) is recommended between 18 and 22 weeks of pregnancy. Evidence has been accumulating on the effectiveness of first-trimester anatomy scans. Early mid-trimester fetal scans (eMTSs; 14–17 weeks) may have the advantage of visualization of most organs, hence allowing earlier genetic assessment and decision making. Our aim is to examine the effectiveness of eMTSs in identifying fetal anomalies compared to lMTSs. **Methods**: A retrospective study was conducted based on data from the multidisciplinary prenatal diagnosis clinic in a tertiary center. During the study period (2011–2021), an out-of-pocket eMTS in a community setting was offered routinely to the general population. Women who had previously undergone an eMTS and were later assessed due to a fetal anomaly in our clinic were included in the study. The cohort was divided into two groups according to whether the anomaly had been detected during the eMTS. We then compared the groups for factors that may be associated with anomaly detection in eMTSs. We used t-tests and chi-square tests, for quantitative and qualitative variables, respectively, to determine variables related to eMTS anomaly detection, and logistic regression for multivariate analysis. **Results**: Of 1525 women assessed in our multidisciplinary clinic, 340 were included in the study. The anomaly detection rate of the eMTS compared to the lMTS was 59.1% The eMTS detection rates for specific organ systems were as follows: skeletal, 57%; cardiac, 52%; congenital anomalies of the kidneys and urinary tract (CAKUT), 44%; central nervous system, 32.4%; chest, 33%; and abdominal, 28%. In multivariate analysis, abnormal first-trimester screening (aOR 3.2; 95%CI 1.26–8.08) and multiple anomalies (aOR 1.86; 95%CI 1.02–3.37) were found to be associated with eMTS anomaly detection. **Conclusions**: The eMTS detection rate was nearly 60% and was most accurate in detecting skeletal, cardiac, and CAKUT anomalies. Since the eMTS was community-based, this rate likely reflects a “real-world” scenario. Our findings support consideration of performing an eMTS or first-trimester scan routinely for earlier diagnosis and decision making, as an adjunctive to lMTSs. Future studies will examine the cost-effectiveness of early scans.

## 1. Introduction

The optimal timing during pregnancy of the fetal anomaly scan remains undetermined. Detection rates of fetal malformations in the second-trimester scan, typically performed in the late mid-trimester between 18 and 22 weeks, varies greatly, between 15% and 85% [1], mostly depending on the type of fetal anomaly. For instance, detection rates approach 100% for gastroschisis and omphalocele [2,3], whereas the detection rate for aortic coarctation is reported to be as low as 22.3% [4].

Detecting fetal anomalies earlier in pregnancy offers numerous benefits, including ample time for further investigation and counseling, which can inform decisions regarding invasive diagnostic testing; intrauterine treatments in conditions such as spina bifida, diaphragmatic hernia, or congenital heart defects; continuation of the pregnancy despite the anomaly; or earlier termination of the pregnancy [5]. Furthermore, earlier in the pregnancy, the transvaginal scan approach may be utilized for diagnostic purposes when there are technical difficulties such as in women that are overweight or those with a scarred uterus [6].

According to recent studies, detection rates of first-trimester scans conducted between 11 and 14 weeks, compared to late mid-trimester fetal organ scans (lMTSs), postnatal neonatal examination, or by post mortem examination, are roughly 45% [3,7,8]. Currently, with the availability of advanced genetic screening methods such as cell-free DNA genetic screening, the nuchal translucency (NT) examination has limited benefits in screening for chromosomal defects. Therefore, some have suggested expanding the utility of the NT scan by performing a broader test or an early anatomical scan [9].

An early second-trimester scan performed at 14–17 weeks (eMTS) for early detection of fetal anomalies, in addition to the routine lMTS, has been offered to women in Israel for many years. However, insufficient data are available regarding fetal anomaly detection rates during the eMTS. It is plausible to assume that performing the eMTS at this time interval would have both the advantage of earlier detection of anomalies than the lMTS and would most likely enable a higher detection rate than the first-trimester scan, as most organs are better visualized. The objective of this study is to examine the effectiveness of eMTSs (14–17 weeks) in identifying fetal anomalies compared to lMTSs.

## 2. Materials and Methods

This was a retrospective analysis that was approved by Shaare Zedek Medical Center institutional review board (0141-24-SZMC) and was exempted informed consent due to its retrospective and de-identified nature. The study included all pregnant women who were referred to our tertiary multidisciplinary fetal diagnostic clinic for further evaluation when a fetal anomaly was suspected in an ultrasound scan between July 2011 and May 2021. The fetal anomaly could have been suspected during NT examination, early or late MTSs, or during a random ultrasound examination performed as part of pregnancy follow-up by either an ultrasound technician or a physician.

Women from the cohort were included in this study only if they had previously undergone an eMTS. We excluded women who had had an eMTS and were referred to our clinic not for a suspected fetal anomaly but rather due to soft markers such as persistent right umbilical vein (PRUV), thickened nuchal fold, or pyelectasis.

The Israeli guidelines recommend an NT scan at 11–13 weeks followed by an lMTS for all pregnant women; both are included in health coverage as directed by the Israel Ministry of Health for members of all health plans. Additionally, an eMTS at 14–17 weeks is offered to all women with an out-of-pocket payment. The guidelines for early and late MTSs are similar. The early scan may be performed by the transabdominal and/or transvaginal approach based on image quality as well as patient and operator preference. Importantly, the eMTS does not replace the routine lMTS, since some anomalies can only be diagnosed at least a month later. The protocol for targeted organ scanning is detailed in Appendix A.

Women in Israel may have their scans performed by various clinics and institutions, and not only by one medical system. All eMTSs and lMTSs are performed by physicians who are trained and certified in ultrasound and fetal anatomical scans. Women evaluated in our multidisciplinary clinic for a suspected fetal anomaly were referred with an ultrasound report, without data regarding the experience and skills of the physician who performed the eMTS.

The assessment in our clinic included a fetal anatomical scan performed by two physicians, each with more than 10 years of experience in performing fetal scans. In cases of any doubt regarding the diagnosis, the images were further reviewed by additional experienced physicians to reach a diagnosis. Additionally, all women had a fetal echocardiogram performed by an experienced fetal cardiologist. When deemed necessary, fetal MRI was performed as well. Women had genetic counseling and were offered amniocentesis and genetic testing utilizing chromosomal microarray analysis. Furthermore, women had counseling by specific disciplines relating to the anomaly detected, i.e., pediatric nephrology consultation when unilateral renal agenesis was found or pediatric neurology when agenesis of the corpus callosum was diagnosed, etc.

The primary outcome of the study was defined as the overall detection rate of fetal anomalies in eMTSs compared to the detailed assessment performed in our clinic (Appendix B).

Secondary outcomes were defined as specific detection rates according to anatomical systems and characteristics of high detection rates.

For this study, fetal anomalies were classified according to organ systems mostly in accordance with the EUROCAT data and as follows: skeletal (including limbs and spine), cardiac (including major vessels), central nervous system, congenital anomalies of the kidneys and urinary tract (CAKUT), abdomen (including abdominal wall defects, abdominal cysts, and retroperitoneal masses), chest, face, and genitalia. When more than one malformation occurred in the same organ system, these malformations were considered as one malformation when calculating detection rates. Abnormal first-trimester chromosomal screening was defined as either NT > 3 mm, detection of a fetal anomaly during the NT examination, or risk assessment for trisomy 21 by first- and/or second-trimester screening equal to or higher than 1/380.

Following identification of women who met the study criteria, we extracted available data regarding demographic and obstetric parameters and details of the fetal anomaly. As patients had their eMTS performed in community-based clinics out of our center and may have opted to continue their care in other institutions after visiting our clinic, we had limited data regarding some of their background demographic and obstetric outcomes.

Statistical analysis. Variables associated with the detection of findings in the early screening were examined by t-tests and chi-square tests, respectively, for quantitative and qualitative variables. Univariate analysis was followed by a multiple logistic regression model. Adjusted odds ratios (aORs) and 95% confidence intervals (CIs) were computed. Statistical analysis was carried out using SPSS software (version 25 statistical package; IBM, Armonk, NY, USA).

## 3. Results

### 3.1. Description of Results

#### 3.1.1. Population Description

A total of 1525 women were assessed at our center between 25 July 2011 and 24 June 2021 due to a suspected fetal anomaly. Of these, 377 women had previously had an eMTS. After excluding 12 women with normal scan results obtained at our center, 18 women referred to us with soft signs, and 7 women with normal neonatal examinations despite abnormal scans, a total of 340 women were included in the study. Patient demographic characteristics are presented in Table 1.

The median gestational age for the early, late, and directed detailed scans was 16.1 weeks [IQR = 15.4–16.5 weeks], 22.4 weeks [IQR = 21.4–23.2 weeks], and 21.6 weeks [IQR = 17.7–24.6 weeks], respectively.

**Figure 1:**
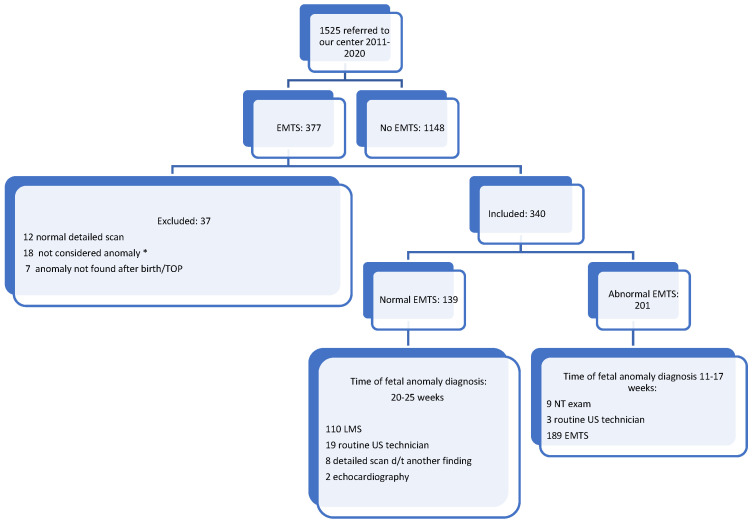
Timing of fetal anomaly detection. * Hydrops, IUGR, akinesia, pedal edema, PRUV, liver calcifications, microcephaly, and macrocrania.

Figure 1 illustrates the timing of anomaly detection. Anomalies identified during the NT scan included megacystis, polydactyly, abdominal wall defect, radial defect, echogenic kidneys, abdominal cyst, and holoprosencephaly. Fetal anomalies detected incidentally by a routine ultrasound scan at 14–15 weeks included encephalocele and gastroschisis. Eight patients were referred for a direct ultrasound scan following an early scan finding that is not considered an anomaly, such as polyhydramnios, oligohydramnios, soft sign, or growth restriction. Further investigation at our center led to the detection of fetal anomalies in these cases. Two patients were referred for fetal echocardiography due to maternal diabetes mellitus or a previous fetus with a cardiac anomaly.

#### 3.1.2. Primary Outcome

Out of the 340 women included in the study, a fetal anomaly was detected in the eMTS in 201 cases (59.1%). The diagnosis rate of anomalies according to gestational age was 55% at week 14, 58% at week 15, 58% at week 16, and 72% at week 17. The *p* value for the trend of detection rate comparing week 15 and week 17 was 0.022. The five most common anomalies were as follows: clubfoot (42 cases), ventriculomegaly (27 cases), digital abnormalities (25 cases), hydronephrosis (22 cases), and interventricular septal defect (17 cases). The diagnostic rates for clubfoot, digital abnormalities, and interventricular septal defect in eMTSs ranged from 71% to 76%. In contrast, the diagnostic rate for ventriculomegaly was 33.3%.

#### 3.1.3. Secondary Outcomes

Specific detection rates according to anatomical systems are detailed in Table 2; the highest detection rate in the eMTS was observed in skeletal anomalies, reaching 57.7%, and the lowest was in abdominal anomalies (28.2%).

#### 3.1.4. Univariate and Multivariate Analysis

In univariate analysis, women with identified anomalies in their eMTS compared to those with negative eMTSs had significantly higher rates of abnormal NT, abnormal first-trimester serum screening, multiple-system anomalies, lethal anomalies, and higher pregnancy termination rates (Table 3). Multivariate analysis demonstrated (Table 4) that abnormal first-trimester screening and a finding of multiple defects are independently associated with a positive eMTS, adjusted OR (aOR) 3.2 (95%CI 1.27–8.08) and aOR 1.86 (95%CI 1.02–3.38), respectively.

## 4. Discussion

In the current study, we examined the detection rate of fetal anomalies during the early second trimester (14–17 weeks). We found that the overall anomaly detection rate during eMTSs performed in a real-world, community-based setting is 59.1%. The highest detection rates were in the skeletal, cardiac, and CAKUT systems, while anomalies in the chest, nervous systems, and abdomen were not detected at a sufficient rate. Factors associated with early detection include an abnormal first-trimester screening (nuchal translucency and/or biochemical markers) and multisystem anomalies and lethal anomalies. Multivariate analysis revealed that abnormal first-trimester screening and multisystem anomalies are independently associated with early mid-trimester positive scans. Furthermore, early detection was associated with higher rates of termination of pregnancy.

Scant literature exists regarding the detection of fetal anomalies in the population by various examiners, during these weeks of the early mid-trimester. A study by LIM et al. (2013) [10] conducted screening between weeks 12 and 17 (average 15 weeks) in women at increased risk for fetal anomalies. A complete anatomy survey could be achieved in 67% of cases, with the heart and the spine having the lowest completion rates. Seventy-two percent of fetal anomalies could be correctly identified. Ebrashy et al. [11] examined the efficacy of early screening between weeks 13 and 14 in a low-risk population and showed a detection rate of 67%. In this study, the success rate of the transabdominal approach was 64%, compared to 82% in the transvaginal approach. The diagnosis rate of anomalies at the end of the first trimester varies significantly among studies. However, when examining recent studies with a substantial sample size, the overall diagnostic rate ranges between 27 and 43% [8,12]. Limiting Singelaki et al.’s study to non-chromosomal abnormalities may reduce the detection rate [12].

Our finding that a positive eMTS was associated with higher rate of termination of pregnancy as opposed to those diagnosed later in the pregnancy (55% vs. 17%, *p* = 0.002) has been reported before [13,14,15]. Several reasons have been postulated to be the cause for this phenomenon: earlier diagnosis may be a sign of a more complex anomaly, it may be associated with a genetic origin of the anomaly, it may enable an earlier and safer termination of the pregnancy, and it may impose less attachment and psychological stress due to the termination [16,17,18,19].

We found that the detection rate of congenital heart defects (CHDs) in eMTSs in community-based centers was 52.5%, while the remaining 47.5% of heart defects were diagnosed in lMTSs. Pike et al. [20] report a 100% detection rate of CHDs by echocardiography performed by an experienced physician between weeks 12 and 16, compared with lMTSs. Yagel et al. [21] report a 40.9% and 98.2% organ visualization rate in the translucency scan compared to eMTSs, respectively, and similar rates for the cardiac outflow tract. In both studies, the high detection rate is likely related to the skill of the physician performing the scan. A meta-analysis by Karim et al. [22] on the detection rate of CHDs between weeks 11 and 14 in a low-risk population, including 45 studies with a total of over 300,000 fetuses, found a rate of 56%, similar to our rate of 51.3%.

The diagnostic rate of abdominal wall defects, such as omphalocele and gastroschisis, was 100% [5/5], similar to other studies [2,3,8]. In contrast, the detection rate of abdominal cysts (such as ovarian, adrenal, and hepatic cysts) was 7.6%. Intra-abdominal cysts appear later in pregnancy, making their identification more challenging at early stages. The diagnostic rates for additional anomalies in the pelvis, hepatobiliary system, and intestines were also very low, despite their high visualization rate at the end of the first trimester [21]. These findings are consistent with diagnostic rates in previous studies [8,23].

We found a 57.7% detection rate of skeletal anomalies in eMTSs, similar to rates described by Vayna, Chen, and Liao (71%, 56–67%, and 33.8%, respectively [2,3,8]). Despite high detection rates of spinal anomalies described in the literature (44% and 94–99% in the first and early second trimester, respectively) [10,21], the four spinal anomalies were not detected in our group. However, we found high detection rates of extremity anomalies: 76% for anomalies of fingers, concurring with previous publications [2,3,8,24]. Clubfoot was diagnosed in our study at a rate of 71%. It seems that the diagnostic rate of clubfoot significantly improves between the first and second trimesters [2,8].

The detection rate of CAKUT anomalies in eMTSs was 45%, which surpasses the 25–34% reported during the first trimester [6,8]. We found a 62% detection rate for lethal CAKUT anomalies. Our eMTS detection of CNS anomalies was 32%, which is low even compared to CNS anomaly detection rates described in the first trimester, which are 50–66% [6,8]. This may be related to the fact that in the first trimester, most anomaly scans are performed transvaginally, while only a few of the eMTSs in the present study were performed transvaginally. No cases of corpus callosum abnormality were detected [0/15]. This is consistent with data that agenesis of the corpus callosum can only be detected from 16 to 18 weeks [23]. Our detection rate of 66% for neural tube defects resembles Liao’s report of 58% [8]. Dandy–Walker malformation may enhance the detection rates for these anomalies [25].

Facial anomalies were identified at a rate of 35%, similar to the 31% detection rate reported by LIAO [8]. The detection rate was 63% for cleft lip and palate and 16% for micro/retrognathia. Cases of hypertelorism and abnormal nasal bone were not diagnosed at all. A study by Lamanna et al. [26] showed that the presence of a genetic problem increased the diagnosis rate of facial anomalies from 50% to 85%.

Based on our analysis, the presence of multiple anomalies increases their detection during eMTSs: 52% in cases of isolated malformations vs. 74% for multiple anomalies. This observation clearly suggests that a single anomaly is more prone to oversight compared to recognizing multiple defects.

Intuitively, as gestational age progresses, the ability to diagnose anomalies is higher. This fact was exemplified in Rossi’s work [6], where an increase in the diagnosis rate was observed, from around 45% at 11 weeks to over 70% at 14 weeks. In our study as well, we observed an increase in the detection rate with the advancement of pregnancy, although it was a moderate increase.

In our study, an abnormal first-trimester Down syndrome screening was significantly associated with a higher detection rate of fetal anomalies in the eMTS. It is well known that positive first-trimester Down syndrome screening is associated with the presence of congenital anomalies [27,28]. This conclusion is supported by Lamanna’s report and was described earlier [27]. It is also well known that some malformations can be detected during the nuchal translucency ultrasound [28]. In addition to these, it is plausible that awareness of the abnormal first-trimester screening increased the index of suspicion to identify anomalies in the eMTS. Furthermore, when first-trimester screening is abnormal, there is an association with genetic disorders that often accompany multisystemic anomalies, which are also detected at a higher rate in the eMTS, compared to isolated anomalies [29].

Recently, a first-trimester scan has been adopted by some professional societies. ISUOG (2013) recommends performing a basic anatomical survey in the first trimester during the nuchal translucency scan [30]. It specifies exactly what organs should be visualized, but organs such as the kidneys, urinary bladder, and even the four chambers of the heart are considered optional.

The strengths of this study include its large sample size, low-risk [“real life”] community-based setting, a standardized protocol for the scan, and a more advanced gestational age than early scans previously described. The study assesses the yield of the eMTS in a real-life setting and in which examiners from the community setting follow a predefined scan protocol. Aside from a small subset of 33 women with abnormal first-trimester Down syndrome screening, most women were from a low-risk population.

Limitations of the study include its retrospective design and partial follow-up data. Postnatal data were available for only 39% of women for confirmation of screening findings. This was due to a combination of missing data, loss of patient follow-up, and pregnancy terminations following the diagnosis of anomalies, which often lacked post mortem analysis. As a result, the assessment of the true findings in the early screening was made in comparison to the targeted lMTS and not to the postnatal data. Therefore, we were unable to describe the predictive value of early MTSs. We are currently collecting data prospectively and plan to describe the predictive value of eMTSs in our next study. In the meantime, we find the 60% detection rate of early MTSs compared with the targeted lMTS to be clinically useful. Only one-quarter of women assessed for fetal anomaly in our center had previously had an early mid-trimester scan, which may not represent our entire population.

Our database lacks information on body mass index, which could have affected the detection rate in the early screening.

Additionally, eMTSs are performed by various clinics and institutions and not only by one medical system. Women evaluated in our multidisciplinary clinic for suspected fetal anomaly were referred with an ultrasound report, without data regarding the experience, skills of the physician who performed the eMTS, or the number of scans performed by each physician, and whether the scan was performed via transvaginal or abdominal approaches.

The study spanned a period of 10 years, during which the detection rate in the early screening may have improved due to advancements in ultrasound technology.

## 5. Conclusions

In our real-life scenario, an eMTS (14–17 weeks) that was performed on a mostly low-risk population by various sonographers detected nearly 60% of fetal anomalies, which is higher than what is generally reported in the first-trimester anatomy scan. Factors associated with higher detection rate in the eMTS include abnormal first-trimester screening and multiple anomalies. Indeed, some of the anomalies that were not detected may not be amenable to detection during the first-trimester scan or the eMTS given their nature (i.e., ACC and ovarian cysts). Advanced gestational age was associated with a higher detection rate, and detection earlier in the gestation was associated with a higher rate of terminations; hence, it is important to redefine the optimal timing for early screening. In an era when non-invasive prenatal testing (NIPT) use is increasing and there has been a suggestion to substitute the nuchal translucency scan with a first-trimester scan for detection of fetal structural anomalies, advantages and disadvantages of this suggestion should be discussed prior to adoption of this paradigm. Our findings suggest that if indeed a first-trimester anatomy scan were adopted, this should be conducted in the interest of earlier prenatal diagnosis, allowing for faster decision-making processes. Yet, given our overall higher detection rate than that described for the first trimester, we propose that the first trimester scan should only be adopted as an adjunctive to the well-established late mid-trimester scan. Future studies are needed to compare scans during the first trimester, eMTSs, and lMTSs.

## Figures and Tables

**Table 1:** Patient demographic characteristics.

Characteristic		N/Total (%)
Age	[mean, range]	30.1 (17.50)
	>35 years	77/340 (22.6)
	>40 years	15/340 (4.4)
Religion	Jewish	297/340 (87.3)
	Muslim	43/340 (12.7)
Parity	Nulliparous	92/320 (28.8)
	Multipara	228/320 (71.2)
Consanguinity		27/312 (8.6)
Previous fetus/child with anomaly		36/305 (11.8)
Congenital defect/genetic abnormality in one or both parents		41/304 (13.4)
IVF/fertility treatments		20/195 (10.2)
Twin pregnancy		17/340 (5)
Abnormal first-trimester screening		33/340 (9.7)
Pregnancy termination		71/244 (29.1)
Fetal anomaly	Multisystem	72/340 (21.2)
	Lethal	17/340 (5)
Anomaly confirmed after birth		133/340 (39.1)

**Table 2:** Diagnostic rates according to various anatomical systems.

Anatomical System	Number of Anomalies Diagnosed out of Total Number of Anomalies	Diagnostic Percentage in Early Screening
Skeletal	60/104	57.7
Cardiac	31/59	52.5
CAKUT	49/109	44.9
Facial	11/31	35.4
Thorax	9/27	33.3
Central nervous system	37/114	32.4
Abdomen	11/39	28.2

**Table 3:** Univariate analysis comparing patients with identified anomalies vs. those with no identified anomalies in early screening.

Characteristic		Normal eMTS % (N)	Abnormal eMTS % (N)	*p*-Value
Age (mean)		30.27	30.02	0.68
Religion	Jewish	42.8 (127)	57.2 (170)	0.06
	Muslim	27.9 (12)	72.1 (31)	
Parity	Nulliparous	40 (40)	60 (60)	0.63
	Multiparous	42.9 (93)	57.1 (124)	
Consanguinity		25.9 (7)	74.1 (20)	0.07
History of previous anomaly		56.3 (9)	43.8 (7)	0.34
	IVF/fertility treatment	34.5 (10)	65.5 (19)	0.31
	Twin pregnancy	47.1 (8)	52.9 (9)	0.59
Abnormal nuchal translucency		13.6 (3)	86.4 (19)	0.001
Abnormal first-trimester biochemical markers		25 (5)	75 (15)	0.02
Abnormal first-trimester screening (NT or biochemical markers)		18.2 (6)	81.8 (27)	0.005
Termination of pregnancy		23.6 (17)	76.4 (55)	0.002
Multisystem anomalies		26.4 (19)	73.6 (53)	0.005
Lethal anomalies		17.6 (3)	82.4 (14)	0.05
Abnormal genetic finding		28.9 (13)	71.1 (32)	0.89

**Table 4:** Multivariate logistic regression analysis for factors associated with positive eMTSs (adjusted odds ratio).

	aOR	95%CI
First-trimester screening	3.2	1.267–8.081
Multisystem anomaly	1.86	1.024–3.378
Lethal anomaly	3.2	0.089–1.164

## Data Availability

The raw data supporting the conclusions of this article will be made available by the authors on request and following institutional IRB approval.

## Appendix A

Protocol for targeted organ scanning

The protocol for targeted organ scanning as specified by the local guidelines includes the following organs: transverse brain view: skull outline, cerebellum, cisterna magna, choroid plexus and lateral ventricles, midline, lips, eyes, lungs, four-chamber view, origin of the great vessels, stomach, kidneys, urinary bladder, umbilical cord abdominal insertion, spine, upper-extremity long bones, existence of hands and feet, number of blood vessels in the umbilical cord, placental location, amount of amniotic fluid, fetal viability, placental location, and if an anomaly was detected, then a detailed scan of the organ with the anomaly [31].

## Appendix B

Protocol for fetal assessment in the fetal anomaly clinic

At our tertiary center, patients who are referred due to a suspected fetal anomaly are scanned by an obstetrician specializing in ultrasonography with at least 10 years of experience performing anatomy scans. The scan is usually performed transabdominally, and a transvaginal scan is performed if the transabdominal scan is not technically adequate, such as in cases with a thickened maternal abdominal wall. The mean time allocated for such scans is 60 min. Most patients, excluding patients who did not consent to full evaluation, also had fetal echocardiography and genetic counseling on the same day, following the detailed scan. Maternal demographic data, medical histories, US findings, and results of chromosomal screening were collected from medical records.

## Appendix C

Fetal anomaly list according to systems and detection rates at first anatomy scan and later detailed scan.

**Table A1:** Specific anomalies detection rate by systems.

Diagnoses by Organ/System (*n* = 495)	Detected at eMTS (213)	Not Diagnosed at eMTS	Detection Rate at eMTS
Skeletal system (104)			**58%**
Scoliosis (2)	0	2	ND
Caudal regression sequence (2)	0	2	ND
Digital irregularity (25)	19	6	176%
Clubfoot (42)	30	12	71%
Absent bone (6)	4	2	66.6%
Arthrogryposis (4)	2	2	50%
Others (23)	5	18	27%
**Cardiac anomalies (59)**			**52.5%**
VSD (17)	13	4	76%
Hypoplastic heart (9)	9	0	100%
TOF (6)	2	4	33%
TGA (8)	3	5	37%
DORV (4)	1	3	25%
Overriding aorta (3)	1	2	33%
Valve anomalies (4)	1	2	33%
AV canal (2)	1	1	50%
Truncus arteriosus (2)	0	2	ND
RV hypertrophy (2)	0	2	ND
Asymmetrical heart ventricles (1)	0	1	ND
Rhabdomyoma [1]	0	1	ND
**Congenital kidney and urinary tract anomalies [CAKUT] (109)**			**45%**
Hydronephrosis (22)	3	19	14%
Ureterohydronephrosis (6)	1	5	17%
MCDK (10)	5	5	50%
Polycystic kidneys (3)	2	1	67%
Echogenic kidney (7)	3	4	43%
Enlarged kidney (5)	3	2	60%
Crossed fused kidney (5)	2	3	40%
Double collecting system (5)	1	4	20%
Unilateral renal agenesis (10)	9	1	90%
Bilateral renal agenesis (2)	2	0	100%
Pelvic kidney (13)	6	7	46%
Horseshoe kidney (7)	7	0	100%
Primary megaureter (1)	0	1	ND
PUV (7)	2	5	28%
Urinary Bladder agenesis (1)	1	0	100%
Bladder exstrophy (3)	1	2	33%
Megacystis (2)	1	1	50%
**Central nervous system (114)**			**33%**
Ventriculomegaly (27)	9	18	33%
Dilated fourth ventricle (2)	1	1	50%
IVH (3)	0	3	ND
Hydrocephalus (3)	3	0	100%
Encephalocele (5)	4	1	80%
Neural tube defect (12)	8	4	67%
Dandy–Walker malformation (3)	1	2	33%
Cerebellum abnormality (11)	3	8	37%
Vermian dysgenesis (7)	2	5	28%
Mega cisterna magna (3)	0	3	ND
Posterior fossa abnormality without Dandy Walker (3)	2	1	67%
Brainstem anomaly (1)	0	1	ND
Arachnoidal cyst (2)	1	1	50%
Interhemispheric cyst (2)	0	2	ND
Periventricular pseudocyst (1)	0	1	ND
Corpus callosum abnormality (15)	0	15	ND
Alobar holoprosencephaly (2)	2	0	100%
Rhomboencephalosynapsis (1)	1	0	100%
Lissencephaly (1)	0	1	ND
Opercular dysplasia (3)	0	3	ND
Colpocephaly (2)	0	2	ND
Prefrontal edema (2)	0	2	ND
Meckel Gruber syndrome (1)	0	1	ND
Dilatation of pericerebral space (1)	0	1	ND
Cloverleaf skull (1)	0	1	ND
**Abdominal anomalies (39)**			**28%**
Esophageal atresia or small/absent stomach (8)	2	6	25%
Gastric septum (1)	0	1	ND
Pyloric stenosis (1)	1	0	100%
Duodenal atresia (1)	0	1	ND
Small bowel dilatation (3)	0	3	ND
Dilated colon (1)	0	1	ND
Anal atresia (2)	1	1	50%
Abdominal cyst (5)	1	4	20%
Meconium pseudocyst (1)	0	1	ND
Splenic cyst (3)	0	3	ND
Adrenal cyst (1)	0	1	ND
Ovarian cyst (2)	0	2	ND
Adrenal hemorrhage (1)	0	1	ND
Hepatic cyst (1)	0	1	ND
Gall bladder agenesis (3)	1	2	33%
Omphalocele (4)	4	0	100%
Gastroschisis (1)	1	0	100%
**Thoracic anomalies (27)**			**33%**
Pleural effusion (2)	2	0	100%
Hydrothorax (7)	3	4	43%
CPAM (12)	2	10	17%
Diaphragmatic hernia (5)	2	3	40%
Bronchial atresia/congenital lobar emphysema (1)	0	1	ND
**Face and neck anomalies (31)**			**35%**
Cleft lip/palate (11)	7	4	64%
Midface hypoplasia (1)	1	0	100%
Hypertelorism (1)	1	0	100%
Hypotelorism (5)	0	5	ND
Abnormal nasal bone (4)	0	4	ND
Macroglossia (1)	0	1	ND
Micrognatia/retrognatia (6)	1	5	17%
Nuchal blebs (2)	1	1	50%
**Genital anomalies (15)**			20%
Cloacal dysgenesis (3)	1	2	33%
Ambiguous genitalia (2)	2	0	100%
Hypospadias/epispadias (6)	0	6	ND
Complete situs invertus (4)	0	4	ND

VSD—ventricular septal defect; TOF—tetralogy of Fallot; TGA—transposition of great arteries; DORV—double outlet right ventricle; AV—atrioventricular; MCDK—multicystic dysplastic kidney; PUV—posterior urethral valve; IVH—intraventricular hemorrhage; CPAM—congenital cystic adenomatoid malformation.
